# Novel *Escherichia coli* RNA Polymerase Binding Protein Encoded by Bacteriophage T5

**DOI:** 10.3390/v12080807

**Published:** 2020-07-26

**Authors:** Evgeny Klimuk, Vladimir Mekler, Darya Lavysh, Marina Serebryakova, Natalia Akulenko, Konstantin Severinov

**Affiliations:** 1 Institute of Molecular Genetics, Russian Academy of Sciences, 123182 Moscow, Russia; jonikl@gmail.com (E.K.); lavyshd@gmail.com (D.L.); 2 Skolkovo Institute of Science and Technology, Center of Life Sciences, 121205 Skolkovo, Russia; 3 Waksman Institute for Microbiology and Department of Molecular Biology and Biochemistry, Rutgers, State University of New Jersey, Piscataway, NJ 08854, USA; mekler@waksman.rutgers.edu; 4 Institute of Gene Biology, Russian Academy of Sciences, 119334 Moscow, Russia; mserebr@mail.ru

**Keywords:** bacteriophage T5, RNA polymerase, transcription regulation, promoter

## Abstract

The *Escherichia coli* bacteriophage T5 has three temporal classes of genes (pre-early, early, and late). All three classes are transcribed by host RNA polymerase (RNAP) containing the σ^70^ promoter specificity subunit. Molecular mechanisms responsible for the switching of viral transcription from one class to another remain unknown. Here, we find the product of T5 gene *026* (gpT5.026) in RNAP preparations purified from T5-infected cells and demonstrate in vitro its tight binding to *E. coli* RNAP. While proteins homologous to gpT5.026 are encoded by all T5-related phages, no similarities to proteins with known functions can be detected. GpT5.026 binds to two regions of the RNAP β subunit and moderately inhibits RNAP interaction with the discriminator region of σ^70^-dependent promoters. A T5 mutant with disrupted gene *026* is viable, but the host cell lysis phase is prolongated and fewer virus particles are produced. During the mutant phage infection, the number of early transcripts increases, whereas the number of late transcripts decreases. We propose that gpT5.026 is part of the regulatory cascade that orchestrates a switch from early to late bacteriophage T5 transcription.

## 1. Introduction

**Figure 1:**
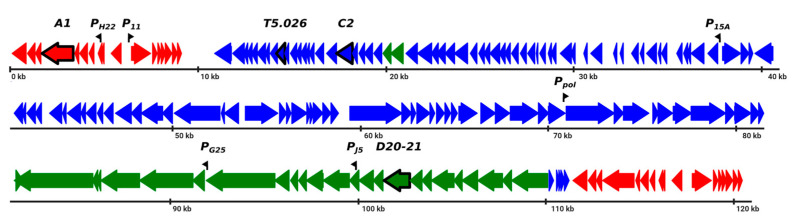
Scheme of the phage T5 genome. Phage genes are shown as colored arrows whose directions match the direction of transcription: red = pre-early genes (located in “first step of transfer” (FST)-DNA in the left upper part of the figure), blue = early, green = late genes. The FST-DNA is duplicated form terminal repeats of the genome. Genes and promoters described in this study are highlighted.

The *Escherichia coli* bacteriophage T5 injects its genome into the host in two steps [1]. First, approximately 8% from one end of the linear double-stranded T5 genome, which is called FST-DNA (from the “first step of transfer”), is injected [1] (Figure 1). FST-DNA is transcribed by host RNA polymerase containing the σ^70^ subunit, leading to production of pre-early viral transcripts. The products of pre-early viral genes initiate the entry of SST-DNA (from the “second step of transfer”) into the cell [2]. SST-DNA is transcribed in two waves, which are referred to as early- and late-phage transcription [3] (Figure 1). Strong σ^70^ promoters have been found in front of both early and late T5 genes [4,5,6,7,8,9]. This observation leads to a question of how coordinated the activation of late genes at the late stage of infection, as well as the inhibition of pre-early and early genes transcription at, respectively, early and late stages of infection, are achieved. 

In earlier studies, attempts to answer this question were made by identification and characterization of phage proteins associated with the host RNAP complex. Pre-early viral proteins at 60 kDa and 11 kDa [10,11,12,13,14], as well as 90 kDa and 15 kDa early proteins [15,16] were shown to co-purify with host RNA polymerase (RNAP) during sedimentation experiments, using infected cells extracts. These early experiments were performed in the absence of T5 genomic sequence information, and the 60 kDa and 90 kDa proteins were assumed to be the products of viral genes *A1* and *C2* [17] (Figure 1), respectively, because they were absent in RNAP preparations from cells infected with phages harboring mutations in these genes. At least for *C2*, the mapping must be wrong, since the *C2* mutation has been mapped to an open reading frame coding for a putative Ser/Thr protein phosphatase with a predicted molecular weight of 32 kDa (Figure 1). Therefore, involvement of the product of phage *C2* gene in the regulation of host RNAP specificity is likely indirect. 

T5 proteins associating with host RNAP proteins were not studied functionally. Analysis of these proteins and the consequences of their interaction with the host transcription apparatus may shed light into the temporal control of T5 gene expression. Here, we report that a product of T5 gene *026* (gpT5.026) binds host RNAP with high affinity (Figure 1). We characterize the gpT5.026 interaction site on RNAP and demonstrate that this protein modulates RNAP activity in vitro. Finally, we show that a T5 mutant lacking gene *026* is defective in a switch from early to late viral transcription. We propose that gpT5.026 is responsible for temporal control of T5 gene expression. 

## 2. Materials and Methods 

### 2.1. Bacterial Strains, Phage and Plasmids

Wild-type *E. coli* K-12 W3110 (F- lambda- IN(rrnD-rrnE)1 rph-1) strain and amber mutation suppressing strains *E. coli* CR63 (serU60(AS) lamB63) and *E. coli* JF238 (F-, ara-55, (lac)3, gyrA91, relA1, spoT1 CGSC) were used for the *wt* T5 phage and T5 phage with amber mutation in gene T5.026 propagation.

*E. coli* XL10-Gold (Δ (mcrA)183 Δ(mcrCB-hsdSMR-mrr)173 endA1 supE44 thi-1 recA1 gyrA96 relA1 lac Hte [F′ proAB lacIq ZΔM15 Tn10 (Tetr ) Amy Camr]) ultracompetent cells (Stratagene) were used for molecular cloning [18]. 

*E. coli* B BL21*(DE3)* (F- dcm ompT hsdS(rB- mB-) gal λ(DE3)) (Stratagene) was used for recombinant protein overproduction.

All bacterial strains were grown in LB media (1% Bactotryptone, 1% NaCl, 0.5% yeast extract, with or without 1.5% Bactoagar) at 37 °C with appropriate antibiotics.

### 2.2. Isolation of RNAP Core

For RNAP purification, the *E. coli W3310* culture was infected with bacteriophage T5 at multiplicity of infection of 10. The cell culture (0.5 L) was collected before infection and at 3, 15, and 25 min after infection. The RNAP core enzyme was purified from these cells as previously described [19], with some modifications. Briefly, cell pellets were resuspended in buffer A (50 mM Tris-HCl (pH 8.0), 10 mM ethylenediaminetetraacetic acid (EDTA), 5% (*v*/*v*) glycerol, 1 mM dithiothreitol (DTT), and 300 mM NaCl, 0.3 mg/mL lysozyme) and incubated for 20 min for lysozyme digestion. The cells were then lysed by sonication, and the lysate was centrifuged at 8000× *g* for 30 min. A 10% solution of Polymin P (pH 7.9) was slowly added to the supernatant with constant stirring to a final concentration of 0.8%. Stirring was continued for another 10 min, followed by centrifugation at 12,000× *g* for 15 min. The pellet was thoroughly resuspended in TGED (10 mM Tris-HCl (pH 8.0), 0.5 mM EDTA, 5% (*v*/*v*) glycerol, 0.1 mM DTT), plus 0.5 M NaCl with the aid of a glass rod. The suspension was centrifuged, and the supernatant was discarded. The pellet washing cycle was repeated at least five times, until no protein was detectable in the supernatant. To elute RNAP, the pellet was resuspended in TGED plus 1 M NaCl. The mixture was centrifuged at 12,000× *g* for 30 min. Finely ground ammonium sulfate was slowly added to the supernatant with stirring, to the amount of 0.35 g per 1 mL solution. The pH was adjusted to 7.0–7.5 with 2 M NaOH, and the mixture was incubated overnight. Ammonium sulfate suspension of the Polymin P eluate was centrifuged, and the pellet was resuspended in a 100-fold volume of buffer TGED, and applied on a 1 mL HiTrap Heparin HP column (GE Healthcare) equilibrated with TGED. The column was washed with 10 column volumes of TGED containing 0.3 M NaCl, and the RNAP was eluted with 5 mL of TGED containing 0.6 M NaCl. Pooled column fractions were concentrated to 0.5 mL in Amicon devices with a 100k cutoff, diluted 10-fold with the storage buffer (40 mM Tris-HC1 (pH 7.9), 0.2 M KCI, 50% (*v*/*v*) glycerol, 1 mM EDTA, 1 mM DTT), concentrated to ~1 mg/mL, and stored at −20 °C.

### 2.3. Trypsin Digestion and Mass Spectrometry

The protein spots were excised from the gel and digested with trypsin. Briefly, the gel pieces (1–2 mm^3^) were washed to remove dye, dehydrated with acetonitrile (ACN), and reswelled with 4 µL of digestion solution containing 20 mM ammonium bicarbonate and 15 ng/µL sequencing grade trypsin (Promega, Madison, WI, USA). The tryptic digestion was left at 37 °C overnight, then the peptides were extracted with 10 µL of 10% ACN containing 0.5% trifluoroacetic acid (TFA). Then, 2 µL of each extract was mixed with 0.5 µL 2,5-dihydroxybenzoic acid-saturated solution in 20% ACN containing 0.5% TFA on the stainless steel Matrix Assisted Laser Desorption/Ionization (MALDI) sample target plate, and dried. Mass spectra were recorded on Ultraflex II MALDI-TOF/TOF mass spectrometer (Bruker Daltonics, DE, USA) equipped with an Nd laser (354 nm). The MH+ molecular ions were detected in reflection mode; the accuracy of monoisotopic mass peak measurement was 70 ppm. Spectra were analyzed using the Mascot software (Matrix Science, London, United Kingdom) through the NCBI database. Partial oxidation of methionine residues and propionamidomethylation of cysteine was permitted; up to one missed tryptic cleavage was considered for all tryptic mass searches. Protein scores greater than 87 were considered as significant (*p* < 0.05).

### 2.4. Cloning, Expression, and Purification of gpT5.026

Gene *T5.026* of phage T5 was cloned into the expression vector pET19b using standard genetic engineering techniques [18]. This plasmid was named pET19b-T5.026. 

To get the protein with a kinase A site, the SphI–NdeI fragment of vector pET33b+ was cloned between the corresponding sites of the plasmid pET19b-T5.026. 

GpT5.026 expression and purification was performed as described [20]. Briefly, expression of gpT5.026 was performed in *E. coli BL21(DE3)* cells using 1 mM isopropyl β-D-1-thiogalactopyranosideas an inductor. After three hours of growth at 37 °C after induction, cells were harvested by centrifugation and resuspended in buffer A (20 mM Tris-HCl pH 8.0, 50 mM NaCl, 0.5 mM β-mercaptoethanol, 5% glycerol) with 1 mg/mL lysozyme. As expressed gpT5.026 was segregated into inclusion bodies, after disruption by ultrasonic treatment, the inclusion bodies were resuspended in buffer B (20 mM Tris-HCl pH 8.0, 0.5 M NaCl, 8 M urea, 0.5 mM β-mercaptoethanol, 5% glycerol). After 1 h incubation at room temperature with occasional mixing, the solution was filtered through an Acrodisc 0.45 Syringe filter and applied on a 1 mL chelating HiTrap column (GE Healthcare) charged with Ni^2+^. The column was washed with buffer B containing 50 mM imidazole, and bound proteins were eluted with buffer B containing 250 mM imidazole. Fractions containing gpT5.026 were combined, diluted to a concentration of 1 mg/mL, and renatured by dialysis against 100 volumes of buffer S (20 mM Tris-HCl (pH 8.0), 200 mM NaCl, 0.1 mM EDTA, 1 mM DTT, 10 mM MgCl_2_, 10 μM ZnCl_2_, and 20% glycerol) overnight with two changes of buffer. They were then concentrated on Centricon Centrifugal Filter Units (Millipore) and supplemented with glycerol to the final concentration of 50%, and resulting proteins (at least 95% pure) were stored at −20 °C. The purity of final preparation was accessed by SDS-PAGE (Figure S1).

### 2.5. Far-Western Blotting Analysis

The recombinant gpT5.026 protein was labeled with an isotope ^32^P using the catalytic subunit of protein kinase A (Sigma), as described [20,21,22], followed by a clean-up on Ni–NTA (Qiagen). Far-Western blotting was performed as previously described [21,23]. Briefly, 1 μg of purified proteins (gpT5.026 or gp2) were incubated with 20 units of protein kinase A in PKA buffer (20 mM Tris-HCl (pH 8.0), 150 mM NaCl, 30 mM DTT, 10 mM MgCl_2_) in the presence of 0.4 mCi of γ-[^32^P] ATP at 30 °C for 1 h, followed by a clean-up on Ni-NTA (Qiagen). 1 μg of RNAP core enzymes or individual RNAP subunits (α, β, β’, and σ^70^) were applied as small drops on a Hybond ECL membrane and annealed for 3 min at 50 °C. Then, membranes were blocked in PROB buffer (20 mM 1,4-Piperazinediethanesulfonic acid (PIPES) (pH 7.4), 200 mM KCl, 1 mM DTT, 2 mM MgCl_2_, 10% glycerol, 0.5% Tween-20, 1% nonfat dried milk) for 2 h at room temperature. Afterwards, each piece of membrane was enveloped in a parafilm sack containing 150 μL of radiolabeled proteins solutions with/without added non-labeled proteins (depending on the design of experiment) in PROB buffer and incubated for 2 h at room temperature. Next, membranes were washed three times with 1 mL of PROB buffer and dried at room temperature for 15 min. Results were revealed using a PhosphorImager (Molecular Dynamics).

### 2.6. Bacterial Two-Hybrid System

Plasmids A1–E8 encode a full-size protein CI of phage λ (λCI) fused with fragments of β, β’, or α subunits of *E. coli* RNAP (aa positions of subunits are indicated in Figure 4), under the control of a promoter *lacUV5*. The plasmid pBRαLN encodes the N-terminal domain and the linker of *E. coli* RNAP α subunit (aa 1–248) under the control of tandem promoters lpp and lacUV5. A PCR fragment containing gene *T5.026* was cloned into the pBRαLN plasmid as a C-terminal fusion with the α subunit coding sequence. The resulting plasmid was named pBRαLN-gpT5.026. *E. coli* strain *BN469* contains the F’ episome, which carries the *lacZ* gene under the control of *lac* promoter and phage λ operator OL2. This strain was transformed with pBRαLN-gpT5.026 together with one of the plasmids A1–E8. All further procedures were performed as previously described [24]. Individual transformed colonies were grown overnight in LB medium in the presence of kanamycin (50 μM), ampicillin (100 μM), chloramphenicol (30 μM), and IPTG (50 μM). The overnight culture was inoculated into LB medium with antibiotics and IPTG at the concentrations indicated above and grown to OD600 0.3–0.7. The cells were disrupted, and the extract was used to measure β-galactosidase activity [24,25]. The experiment was repeated four times.

### 2.7. Obtaining T5 Phage with Amber Mutation in Gene T5.026

Amber mutation in gene *T5.026* was obtained by oligonucleotide-directed mutagenesis. At the initial stage, two PCR reactions were performed using pairs of primers: A1 and A2, and B1 and B2 (Table 1). Purified PCR products were mixed and amplified using primers A1 and B2. The resulting PCR product was cloned into the vector pUC19. The plasmid was named pUC19-amT5.026. Suppressor strain *E. coli CR63* was transformed with pUC19-amT5.026 and infected with phage T5, with a multiplicity of infection of 10 plaque-forming units (PFU) per cell. The cell lysate was plated on *E. coli CR63*. In order to identify the amber mutant, plaques were transferred to a nylon membrane (Hybond-N+, GE Healthcare) and analyzed by hybridization with ^32^P-labeled primer A2, according to the manufacturer’s recommendations. The presence of the amber mutation in the gene *T5.026* was confirmed by sequencing.

### 2.8. One-Step Growth Experiment

*E. coli* strain *JF238* was grown at 37 °C in the MGM medium [26] to OD600 = 0.4–0.6 and infected with a multiplicity of infection 10^−5^. Adsorption of the phage was performed for 10 min. Samples were taken at different stages of infection and plated for counting phage particles directly or after appropriate dilution.

### 2.9. Primer Extension Reactions

*E. coli JF238* cells were infected with wild-type or T5*amT5.026* at a multiplicity of infection of 10 and 15 mL of culture at 0, 5, 15, 20, 30, 40, and 50 min after infections were collected. Isolation of total RNA from cells was performed as previously described, with minor modifications [27]. Shortly, cells were resuspended in 0.5 mL of ASE buffer (20 mM Na-acetate, pH 4.8; 0.5% SDS; 1 mM EDTA). The suspension was mixed with an equal volume of acidic phenol, preheated to 60 °C, and incubated for 10 min with constant stirring, followed by separation of the phases by centrifugation. Procedure with the acidic phenol was repeated twice. Then RNA was precipitated with ethanol, the pellet was washed, vacuum-dried, and dissolved in 50 μL of diethyl pyrocarbonate (DEPC)-treated water. Analysis of transcription by primer extension was performed as described [18]. The primer was labeled with ^32^P-ATP by phage T4 polynucleotide kinase (New England Biolabs), as recommended by the manufacturer. One pmol of a mixture of ^32^P-labeled primers and 10 μg RNA in 40 mM PIPES (pH 6.4), 400 mM NaCl, 1 mM EDTA, and 80% formamide were heated at 85 °C for 10 min, followed by overnight incubation at 0 °C. RNA with annealed primer was precipitated with ethanol, washed, dried, and dissolved in water. RNA was reverse-transcribed using M-MuLV (SibEnzyme), according to manufacturer’s recommendations. The reaction products were dissolved in 7 M urea-formamide loading buffer and resolved on 7% polyacrylamide, 7 M urea sequencing gels. Sanger sequencing reactions were performed with the same end-labelled primers using fmol DNA Cycle Sequencing System (Promega), and the products, separated alongside primer extension reactions products, we used as markers. Reaction products were revealed using PhosphorImager (Molecular Dynamics). Quantification of signals from radiolabeled products was performed with ImageJ Software.

### 2.10. Fluorometric Assays 

RNAP holoenzyme containing σ^70^ derivative labeled at position 211, with fluorescent label 5-TMR ([211Cys-TMR] σ^70^), was prepared as previously described [28]. Fluorescence measurements were performed using a Quanta-Master QM4 spectrofluorometer (PTI) in transcription buffer (40 mM Tris-HCl pH 8.0, 100 mM NaCl, 5% glycerol, 1 mM DTT, and 10 mM MgCl_2_) containing 0.02% Tween 20 at 25 °C. Final assay mixtures (800 μL) contained 1 nM labeled RNAP holoenzyme and DNA probes at various concentrations. The TMR fluorescence intensities were recorded with an excitation wavelength of 550 nm and an emission wavelength of 578 nm. Time-dependent fluorescence changes were monitored after manual mixing of RNAP beacon (800 μL) and a DNA probe (<20 μL) in a cuvette; the mixing dead time was 15 s.

Double-stranded and fork junction DNA probes were prepared by the annealing of DNA oligonucleotides synthesized by Integrated DNA Technologies, as described previously [28,29].

To obtain equilibrium dissociation constants (Kd) of the RNAP beacon with oligonucleotides and fork junctions, the experimental dependence of the fluorescent signal amplitude on DNA probe concentration was fitted to a chemical equilibrium equation (i.e., titration assay), as previously described [28]. The Kd values were determined as averages obtained from three independent experiments, and the standard deviations were less than 25% of the corresponding mean values. 

## 3. Results

### 3.1. Detection and Identification of Proteins Associated with host RNAP in T5-Infected Cells 

**Figure 2:**
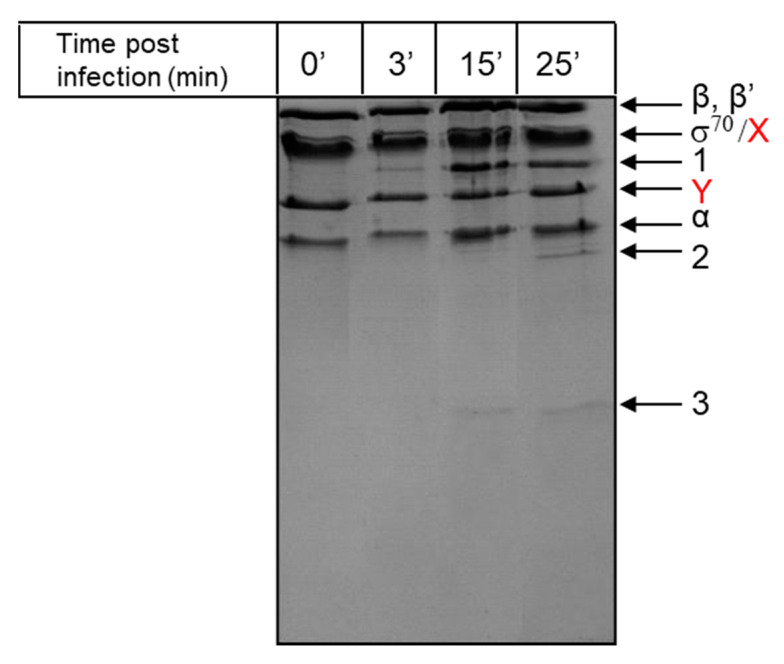
*E. coli* RNA polymerase (RNAP) purified at different stages of infection by bacteriophage T5. RNAP from uninfected cells (0) and at 3, 15, and 25 min post-infection was analyzed by SDS-PAGE electrophoresis and stained with Coomassie G-250. RNAP core subunits α (40 kDa), β (150 kDa), and β’ (155 kDa); the σ^70^ subunit (apparent molecular weight 90 kDa); contaminating proteins X and Y (highlighted in red color font); and proteins present only in preparations of RNAP purified from infected cells, marked as 1, 2, and 3, are shown.

In order to identify transcription factors of the T5 phage, RNAP was purified from cells collected 3, 15, and 25 min post-infection, and proteins present in each preparation were compared to proteins present in RNAP purified from uninfected cells (Figure 2). RNAP from uninfected cells contained the RNAP core subunits (β’ and β, 155 and 150 kDa, respectively; the α subunit (40 kDa); the σ^70^ subunit (70 kDa, migrates as a 90 kDa protein during SDS-PAGE); and two major additional bands (labeled X and Y on Figure 2). These proteins are present in RNAP samples even after the last purification step—heparin–agarose affinity chromatography—and can be removed after the Mono Q ion-exchange chromatography step. Three protein bands (indicated as 1, 2, and 3 in Figure 2) with apparent molecular weights of 60, 35, and ~17 kDa, respectively, were present only in preparations of RNAP from phage-infected cells. Bands 1 and 3 appeared earlier during the infection, and their abundance increased as the infection progressed. Band 2 was only found in RNAP preparation from cells collected late (25 min) in the infection. Proteins of bands 1, 2, and 3 were identified by mass spectrometric analysis as the products of genes *A1*, *D20-21*, and *T5.026*, respectively. GpA1 (60 kDa) was detected in RNAP preparations in early studies of bacteriophage T5 infection [10,14]. GpA1 is thought to be involved in several different processes, such as host DNA degradation and regulation of the transcription of pre-early genes [30,31,32]. GpA1 is also required for the second-step transfer of phage DNA into infected cells [32]. The product of gene *D20-21* is a major head protein (pb8) with a calculated molecular weight of ~50 kDa [33]. This protein is proteolytically processed to a ~32 kDa form that is present in 775 copies per virion [34]. Considering the amount of progeny virions, and thus the abundance of this protein late in infection and the fact that it is only detected in RNAP preparations from cells collected late in infection, the presence of the pb8 fragment in RNAP preparations is most likely due to non-specific interaction. GpT5.026 (calculated molecular weight = 18.178.3 Da, calculated pI = 5.93) is a protein of unknown function; its interaction with host RNAP has not been reported previously. Gene *026*, coding for gpT5.026, is an early gene, located close to the boundary of FST- and SST-DNA (Figure 1). 

### 3.2. Interaction of gpT5.026 with RNAP Core Enzyme

**Figure 3:**
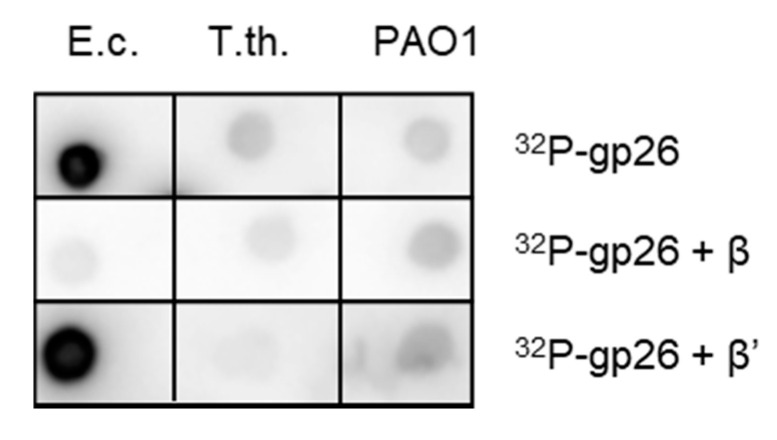
Analysis of the interaction between gpT5.026 and RNAP core enzyme by Far-Western dot blotting experiment. Membranes containing *E. coli* (“E. c.”), *T. thermophilus* (“T. th.”), and *P. aeruginosa PAO1* (“PAO1”) RNAPs were incubated only with ^32^P-labeled gpT5.026 (“^32^P-gpT5.026”), as well as in the presence of *E. coli* β (“^32^P-gpT5.026 + β”) or β’ subunits (“^32^P-gpT5.026 + β’”). The results were visualized by autoradiography using a Phosphorimager.

Both the *A1* gene and the *T5.026* gene were cloned in expression vectors to validate the interaction with host RNAP. Despite numerous attempts and various expression strategies used, we were unable to express the gpA1 protein, which appeared to be highly toxic to *E. coli*. The analysis of this protein was therefore discontinued. Recombinant gpT5.026, N-terminally fused to hexahistidine tag and the protein kinase A phosphorylation site, was expressed in high yield, segregated in inclusion bodies, and solubilized by renaturation in vitro. We tested whether gpT5.026 interacts with host RNAP by Far-Western dot experiment. GpT5.026 was ^32^P-labeled and used to probe with *Escherichia coli*, *Thermus thermophilus*, and *Pseudomonas aeruginosa* RNAP core enzymes spotted on nitrocellulose membrane. After washing, residual radioactivity on the membrane was monitored. As can be seen from Figure 3, radioactive gpT5.026 bound the *E. coli* RNAP core but not *T. thermophilus* or *P. aeruginosa* RNAPs. To determine which RNAP subunit is involved in the interaction, ^32^P-labeled gpT5.026 was pre-incubated with 10-fold molar excess of β or β’ subunits, and these protein mixtures were used to probe membranes with immobilized RNAP. Significant membrane signal reduction was observed when the membrane was probed with a mixture of ^32^P-labeled gpT5.026 and β subunit. In contrast, the presence of a β’ subunit did not affect the signal (Figure 3). 

To directly demonstrate that gpT5.026 interacts with the *E. coli* RNAP β subunit, ^32^P-labeled gpT5.026 was used to probe a membrane containing a spotted RNAP core and individual core subunits: α, β, β’, and σ^70^. As a control, ^32^P-labeled T7 gp2, a protein known to interact with the *E. coli* RNAP β’ subunit jaw domain [35], was used. The results are presented in Figure S2. T7 gp2, as expected, interacted with the RNAP core and the β’ subunit. In contrast, ^32^P-labeled gpT5.026 interacted with RNAP core and β. Excess of unlabeled gpT5.026 strongly decreased labeled gpT5.026 interaction with the core, but had a milder effect on the interaction with ^32^P-labeled β. We conclude that gpT5.026 interacts with *E. coli* RNAP mainly through the β subunit.

**Figure 4:**
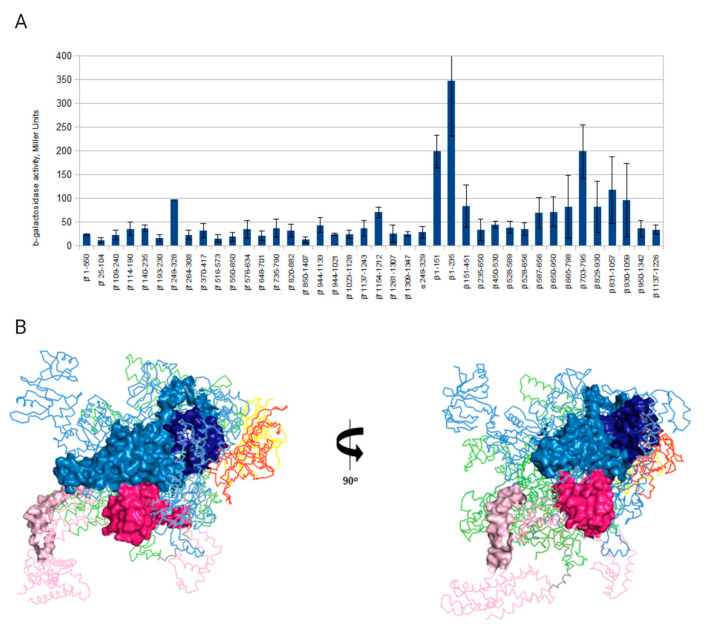
GpT5.026 binding sites on an *E. coli* RNAP holoenzyme. (**A**) Identification of gpT5.026 binding sites on host RNAP subunits with a bacterial two-hybrid assay. Two-hybrid proteins were co-expressed in *E. coli* cells bearing a *lacZ* reporter gene under the control of the *lac*-promoter and O_L_2 operator. One of the hybrid proteins consists of the part of the RNAP α subunit and gpT5.026, while another one is a fusion between CI and fragments of β, β’, or α subunits of *E. coli* RNAP (amino acid positions of these fragments are indicated on the abscissa under corresponding bar). Cells containing hybrid proteins were disrupted, and β-galactosidase activity was measured in crude extracts (described in Materials and Methods). The experiment was conducted in four replicates. Means and standard deviations from the mean are shown. (**B**) Crystal structure of the *E. coli* RNAP holoenzyme at 3.6A° resolution (4LJZ, [37]). The β’, two α, and ω subunits are made transparent for better perception, and are colored green, red/yellow, and grey, respectively. The σ^70^ subunit is shown in pink (regions 1.2 and 3.1 are marked pale pink and dark pink, respectively, and are shown by surface representation) and the β subunit is shown in blue (amino acids 1 to 151 and 703 to 795 are marked pale and dark blue, respectively, and shown by surface representation).

To map gpT5.026 interaction sites on RNAP, a bacterial plasmid-based, two-hybrid assay was used [36]. The pBRαLN-gpT5.026 plasmid encoding the N-terminal domain and the linker of the *E. coli* RNAP α subunit fused to gpT5.026 was constructed. *E. coli lacZ* strain, containing an F’ episome with *lacZ* under control of a *lac* promoter variant fused to phage λ OL2 operator, was transformed with pBRαLN-gpT5.026 together with compatible plasmids expressing a full-sized λ phage CI transcription regulator fused with various fragments of RNAP β, β’, or α subunits. An interaction between the α hybrid encoded by pBRαLN-gpT5.026 and the CI hybrid encoded by a compatible plasmid leads to β-galactosidase production [36]. In agreement with Far-Western analysis presented above, no interaction between the α-gpT5.026 hybrid and β’ or α subunit hybrids was detected (Figure 4A). In contrast, the interaction of α-gpT5.026 hybrid with hybrids containing N-terminal β subunit amino acid residues 1–235 and 1–151, and with a central β subunit part (amino acids 703–795), was detected (Figure 4A). The interaction with the 1–235 part was particularly robust. A β fragment containing amino acids 151–451 did not interact with gpT5.026. Fragments of β from 650–950 and 665–798 (and therefore carrying the entire putative binding epitope between amino acids 703–795, above) showed no interaction with gpT5.026. A possible explanation could be that the central interaction site is masked in longer β fragments. We consider the 703–795 binding site as tentative, while the N-terminal binding site likely constitutes the major site of the interaction. Interestingly, β amino acids 1–151 and 703–795 form a continuous area on the surface of the *E. coli* RNAP (Figure 4B), indicating that they may jointly form a binding site for gpT5.026.

### 3.3. The Role of gpT5.026 in T5 Transcription Regulation in Vivo

**Figure 5:**
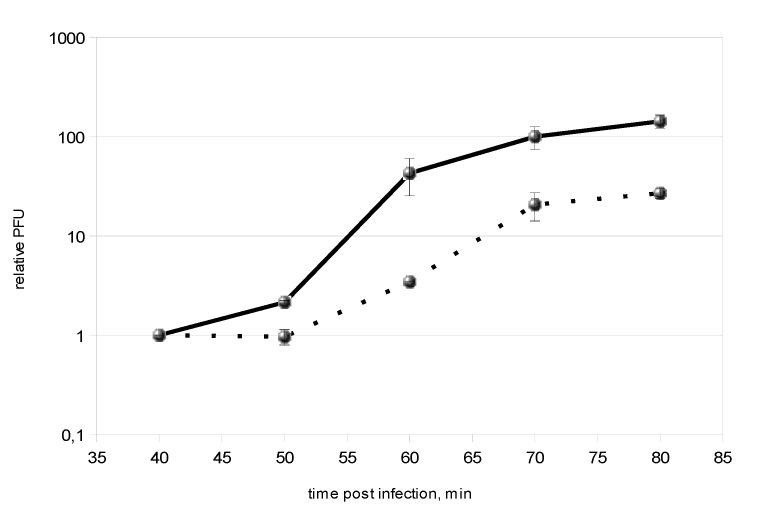
One-step growth curves of phages T5^wt^ and T5*amT5.026*. The *E. coli JF238* culture was infected with wild-type and T5*amT5.026* phages, and aliquots were taken at different minutes after infection and plated in soft agar to determine the number of plaque-forming units (PFU). The ordinate shows the ratio of PFUs at indicated time points of infection to the number of PFUs at 40 min post-infection (relative amount of PFUs); the number of PFUs remains constant up to 40 min. The experiment was repeated three times. Means and standard deviations from the mean are shown. Continuous and dashed lines show the change in the relative amounts of PFU in the course of infection by phages T5wt and T5*amT5.026*, respectively.

To determine the role of gpT5.026 during T5 infection, a mutant phage with an amber mutation in the beginning of the *T5.026* gene was constructed by homologous recombination. A mutant, initially obtained on an amber suppressing host, was able to productively infect a non-suppressing, wild-type *E. coli* cell. We conclude that *T5.026* is not an essential gene. A single-step growth experiment in a rich medium, conducted with wild-type and mutant T5*amT5.026* phages and using non-suppressing cells as a host, revealed that the latent period in both cases was approximately the same (ca. 50 min; Figure 5), but the mutant phage burst size was five times less than that of the wild-type phage (24 versus 120 PFU per infected cell, respectively). We therefore conclude that gpT5.026 impairs infection of the host.

To monitor viral transcription during the wild-type and mutant phage infection, a multiplex primer extension assay was used. Total RNA was prepared from cells collected at various time points post-infection and subjected to primer extension analysis, with primers annealing downstream of T5 promoters, which were previously identified in in vitro transcription [38]. Out of 19 promoters tested [38], primer extension products corresponding to 11 promoters were observed. Primer extension analysis of RNA purified from infected cells, with primers annealing downstream of intergenic regions that could contain additional promoters, revealed four additional primer extension products that contained plausible σ^70^ promoter consensus element in front of them.

**Figure 6:**
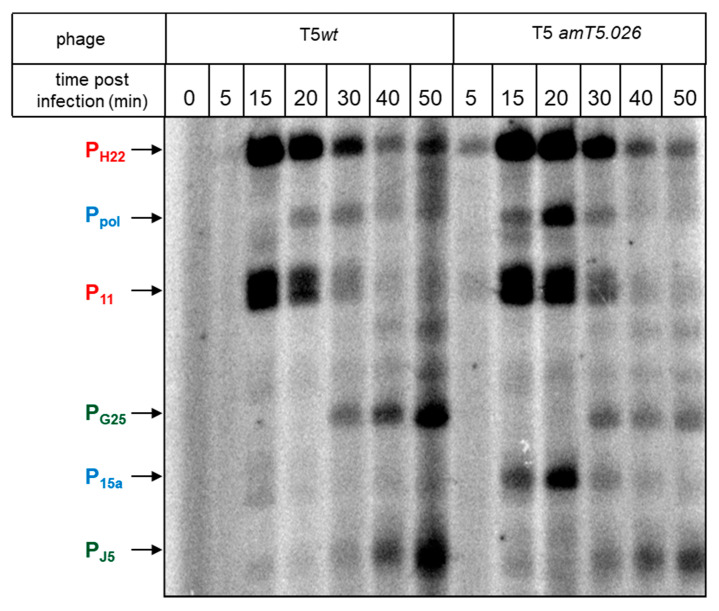
Analysis of transcription in *E. coli* cells infected with phage T5^wt^ and T5*amT5.026*. RNA was isolated from uninfected (0) and infected cells collected 5, 15, 20, 30, 40, and 50 min after the infection. Detection of transcripts from the promoters of different temporal classes was carried out by primer extension assay with a mixture of ^32^P-labeled primers. The reaction products were separated in 7% denaturing polyacrylamide gel and visualized using a Phosphorimager. Primer extension products are indicated by arrows, and the promoters they arise from are labeled (pre-early-P_11_ and P_H22_, early-P_15A_ and P_pol_, and late-P_G25_ and P_J5_ promoters). Quantification of transcript kinetics is shown in Figure S3.

Based on the kinetics of primer extension product accumulation during infection, promoters whose activity profiles corresponded to pre-early, early, and late temporal classes were validated (Figure 6, Figure S3). In the case of wild-type infection, pre-early transcripts from promoters P_11_ and P_H22_ were most abundant 15 min post-infection, and gradually decreased at later times. Early transcripts from promoter P_pol_ appeared 20 min post-infection, and disappeared after 30 min. A primer extension product corresponding to another early promoter, P_15A_, had similar temporal kinetics but was barely visible on the gel. Late transcripts from P_J5_ and P_G25_ appeared 30 min post-infection and accumulated continuously afterwards. A different transcription pattern could be observed in cells infected with the mutant phage. Pre-early transcript kinetics were unchanged. Early transcripts reached a peak 20 min post-infection, and were generally more abundant than corresponding transcripts in the wild-type infection (the effect is clearly seen in the case of the P_15A_ transcript), while the abundance of late transcripts (but not the kinetics of their accumulation) was decreased. These effects were highly reproducible. Quantification of radioactivity in primer extension product bands (Figure S3) corresponding to early and late transcripts indicated that at same time points, the abundance of early transcripts was increased ca. five-fold during the mutant infection, while the abundance of late transcript was decreased by the same amount. Thus, in the absence of gpT5.026, the switch from early to late viral transcription is affected, and the number of late transcripts, which code for viral progeny structural proteins, is decreased. The decrease in transcription of late viral genes, which encode virion proteins, is likely responsible for decreased phage yield observed in mutant phage infections.

### 3.4. Influence of gpT5.026 on the DNA Binding Properties of the σ^70^ RNAP Holoenzyme, and Quantitative Analysis of gpT5.026–RNAP Interaction

The effect of recombinant gpT5.026 on *E. coli* RNAP activity was studied in several in vitro transcription assays, including σ^70^-dependent transcription initiation, transcription elongation, and transcription termination assays. While gpT5.026 appeared to somewhat inhibit abortive transcript synthesis from linear DNA fragments containing some promoters, the effects were generally small (two-fold or less, [39]). No effect on post-initiation activities of RNAP was observed.

**Figure 7:**
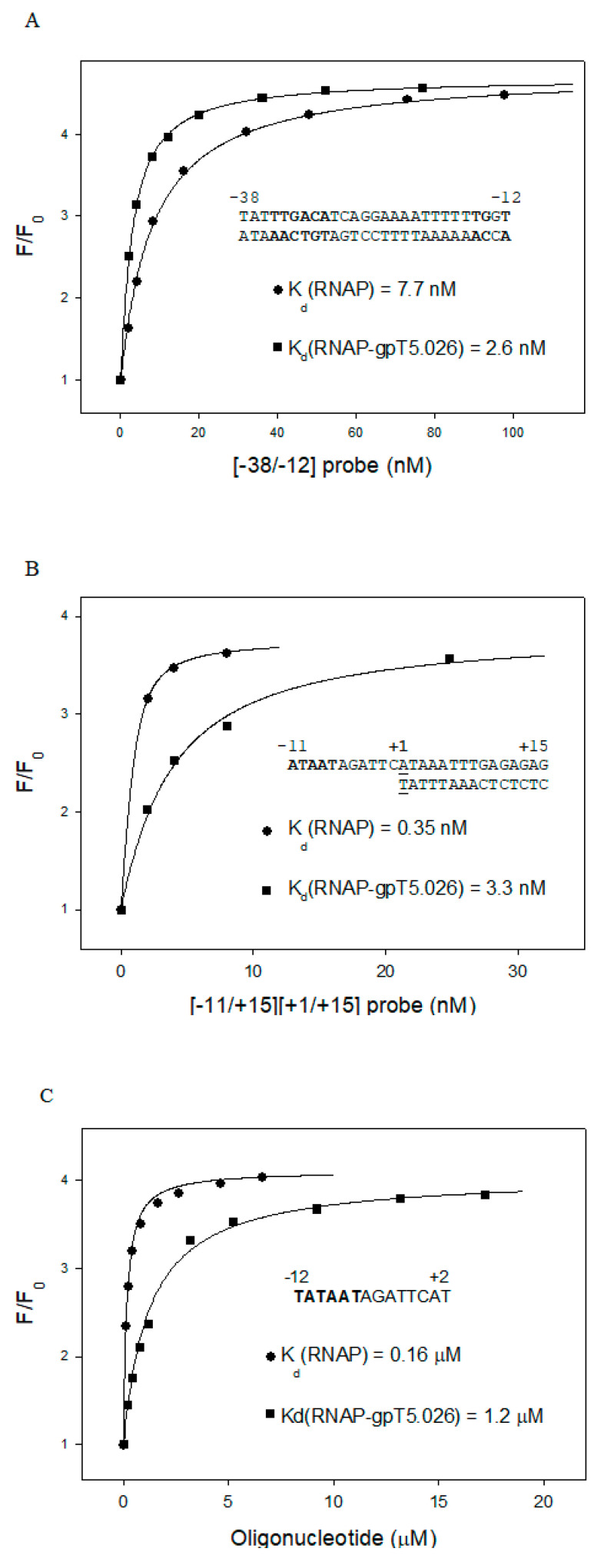
RNAP beacon assays reveal the effect of gpT5.026 on RNAP interactions with model promoter fragments. Relative fluorescence intensity (F/F_0_) change upon titration of [211Cys-TMR] σ^70^ holoenzyme and the [211Cys-TMR] σ^70^ holoenzyme preincubated with gpT5.026 (100 nM) for 15 min with the [−38/−12] upstream double-stranded probe (**A**), the [−11/+15][+1/+15] downstream fork junction (**B**), and the −12/+2 oligonucleotide (**C**). The solid lines correspond to a nonlinear regression fit of the data. In DNA probe names, numbers indicate probe boundaries with respect to the transcription start position, located at +1. Numbers in left and right parentheses in the fork junction probe name correspond to boundaries of the upper and bottom strands of the fork junction probe.

*E. coli* RNAP is known to specifically bind to certain model promoter fragments [28,40,41,42]. RNAP interactions with such DNA probes mimic RNAP interactions with corresponding promoter segments. To dissect the weak effect of gpT5.026 on transcription initiation, we studied the effects of gpT5.026 on RNAP binding to three model promoter fragments (shown in Figure 7), using a highly sensitive fluorescent RNAP beacon assay [28,29]. The assay employs a functional σ^70^ derivative (beacon) with a fluorescent label site specifically incorporated in proximity to region 2.3, the part of σ^70^ involved in the recognition of the −10 promoter element. An RNAP holoenzyme containing labeled σ^70^ has low ground-level fluorescence, because region 2.3 aromatic amino acids quench the fluorescence of the label. After binding to a promoter and establishing specific contacts between region 2.3 aromatic amino acids and the −10 promoter element, the label is “unquenched”, leading to a strong increase in fluorescence. 

We found that gpT5.026 improved the binding of a double-stranded −38/−12 probe by ~3-fold (Figure 7A). Interestingly, RNAP affinity to a downstream fork junction probe that is known to recapitulate functional properties of the transcription bubble and downstream dsDNA of the open promoter complex [42,43] decreased considerably in the presence of gpT5.026, at ~10-fold (Figure 7B). Moreover, gpT5.026 decreased RNAP binding to an oligonucleotide (TATAATAGATTCAT), whose sequence corresponds to positions −12 to +2 of the T5N25 non-template strand in the downstream fork used, by eight-fold (Figure 7C), while it had no effect on the binding of a shorter oligo corresponding to positions −12/−6 [39]. The results thus show that gpT5.026 modifies the DNA binding properties of the σ^70^ RNAP holoenzyme, and suggest that gpT5.026 may affect formation of the open promoter complex by decreasing affinity of the enzyme to the −5/+2 segment of the non-template strand of the transcription bubble.

**Figure 8:**
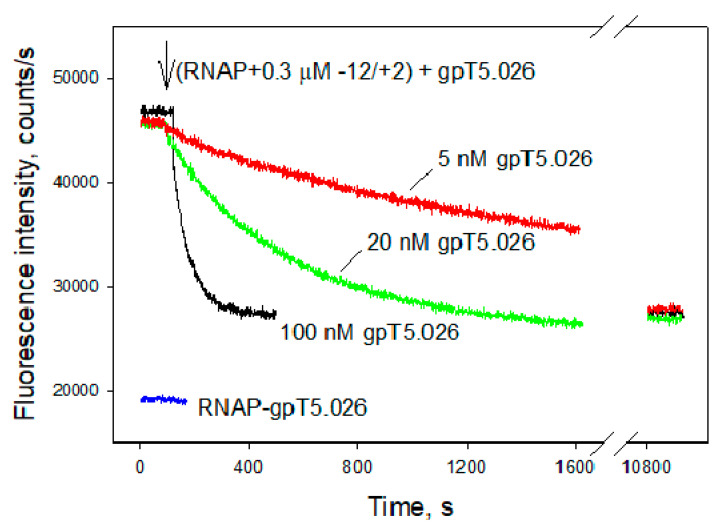
Evaluation of gpT5.026 affinity to RNAP by the RNAP beacon assay. Three samples containing 1 nM [211Cys-TMR] σ^70^ RNAP and 0.3 μM −12/+2 oligonucleotide were incubated for 5 min. Then 100, 20, and 5 nM gpT5.026 were added to the samples, and fluorescence intensity was monitored over time.

The inhibition of the −12/+2 oligonucleotide binding to RNAP was used to evaluate the Kd value of gpT50.26 binding to RNAP. To this end, we measured the signal generated by a 1 nM RNAP beacon in the presence of 300 nM −12/+2 without gpT5.026 and upon the addition of 100, 20, and 5 nM of gpT5.026. As can be seen from Figure 8, the equilibrium signal intensities reached after 3 h incubations were nearly the same in all samples containing gpT5.026. The result implies that the 5 nM gpT5.026 concentration is sufficient to practically completely (>80%) saturate the RNAP–gpT5.026 complex. This sets the high estimate of Kd for the RNAP–gpT5.026 complex at <1 nM. More precise determination of the binding constant was deemed to be impractical, since gpT5.026 concentrations less than 5 nM required very long times to reach the equilibrium binding.

## 4. Discussion

It has been shown in early studies that several proteins bind RNAP in T5-infected cells; however, only one of them (gpA1) has been positively identified based on genomic annotation [10,14]. Here, we find a product of gene *T5.026* in preparations of RNAP obtained from infected cells. GpT5.026 has no homologues among proteins with known function in public databases, although gene *T5.026* was found in many T5-related phages and is highly conserved, not only within the *Tequintavirus* genus but also within the wider *Demerecviridae* family (Figure S4). GpT5.026 is encoded in the region of early genes of T5-like bacteriophages genomes, which makes it a candidate for transcription regulation at later stages of infection. We demonstrate that gpT5.026 binds to the RNAP with high affinity (Kd approximately 1 nM). Two regions of the β subunit (amino acids 1 to 151 and 703 to 795) are involved in these interactions (Figure 4). These regions are positioned close to each other in the protein tertiary structure. GpT5.026 has no strong effect on transcription in vitro, which is consistent with the location of its binding site on the RNAP molecule, away from the DNA and from the σ^70^ regions participating in the interactions with promoter consensus elements. At the same time, the use of a more sensitive beacon assay showed that gpT5.026 moderately inhibits the interaction of RNAP with the promoter DNA −5/+2 (so-called discriminator) region. It was demonstrated earlier that σ^70^ region 1.2 is involved in contacts with a discriminator [44]. Figure 4B shows that this region of σ^70^ is remote from the gpT5.026 binding site. Therefore, gpT5.026 is likely to have an indirect effect on the interaction of RNAP σ^70^ holoenzymes with the discriminator region of a promoter.

A multiple alignment of gpT5.026 homologs from the indicated phages is presented. 

In vivo, the absence of gpT5.026 does not prevent the switch of transcription from early to late genes, but changes the number of transcripts synthesized. Compared to the wild-type phage infection, in the absence of gpT5.026, more early transcripts and fewer late transcripts are synthesized. The lower level of transcription of late genes encoding virion proteins is likely responsible for the observed five-fold decrease in the yield of the mutant phage. 

Alignment of T5 early and late promoter sequences (Figure S5) shows that phage promoters differ mainly by their discriminator region. All early promoters identified to date contain consensus sequence 5′-ATATT-3′ in this region. Probably, this motive is responsible for different effects of gpT5.026 on transcription of early and late genes. It is possible that in vivo phage or host proteins enhance the effect of gpT5.026 on the interaction between the σ^70^ holoenzyme and the discriminator region of promoters. These proteins and their mechanisms of action remain to be identified.

## Figures and Tables

**Table 1:** Primers designed for oligonucleotide-directed mutagenesis in gene *T5.026*.

Primer Name	Nucleotide Sequence (5’-3’)
A1	CCTGAGAAGCTTTTTACAAAATACTCACCATC
A2	ACTGGATAAACTAGTCGTTAAATTGGTTAT
B1	GACTAGTTTATCCAGTTATTTGTCGAAACG
B2	GGTTCCGGATCCGTTGTTAAAAATATTGAAAC

## Supplementary Materials

The following are available online at https://www.mdpi.com/1999-4915/12/8/807/s1, Figure S1: Final preparation of gpT5.026 on 4–20% SDS-PAGE; Figure S2: Analysis of the interaction between gpT5.026 and isolated RNAP subunits by Far-Western dot blotting experiment; Figure S3: Quantification of indicated T5 transcripts seen on the gel shown in Figure 6, using ImageJ software; Figure S4: Conservation of gpT5.026 among phages of Tequintavirus (T5-like) genus and Demerecviridae family; Figure S5: Multiple sequence alignment of early and late promoters of phage T5 [45].
