# Supplementary material for: Novel Escherichia coli RNA Polymerase Binding Protein Encoded by Bacteriophage T5
Source: Viruses. 2020 Jul 26;12(8):807. doi: 10.3390/v12080807 (PMC7472727; doi:10.3390/v12080807)
Supplement: Supplementary file 1 [file viruses-12-00807-s001.pdf]

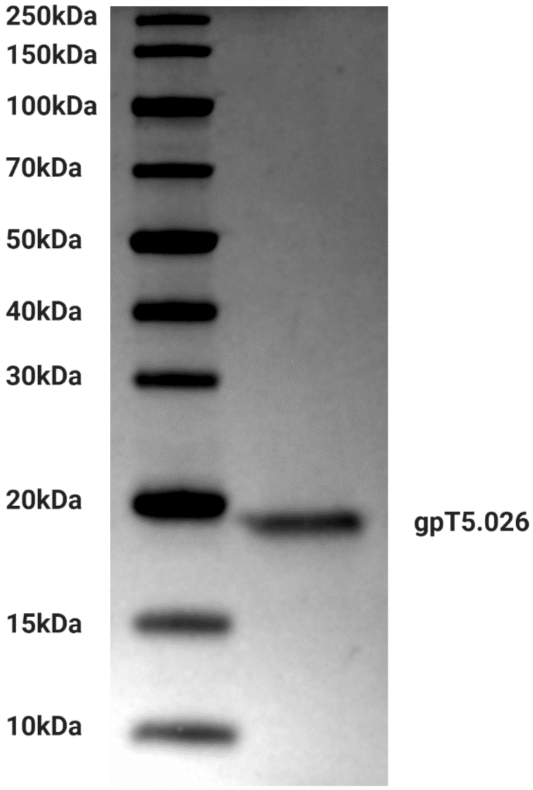

Figure S1. SDS polyacrylamide gel showing the purity of recombinant gpT5.026. A Coomassie-stained 4–20% sodium dodecyl sulphate (SDS) polyacrylamide gel is presented.

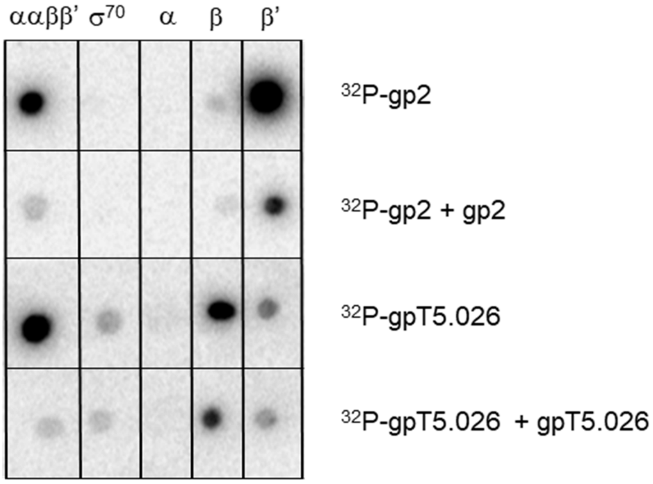

Figure S2. Analysis of interaction between gpT5.026 and RNAP subunits by Far-Western dot blotting experiment. Membranes containing an *E. coli* RNAP core (labeled "ααββ′") and σ^{70}, α, β, and β′ subunits (labelled accordingly) were incubated only with ^{32}P-labeled gp2 of phage T7 ("^{32}P-gp2") or gpT5.026 ("^{32}P-gpT5.026"), as well as in the presence of a 50-fold excess of unlabeled proteins ("^{32}P-gp2 + gp2" or "^{32}P-gpT5.026 + gpT5.026", accordingly). The results were visualized by autoradiography using a Phosphorimager.

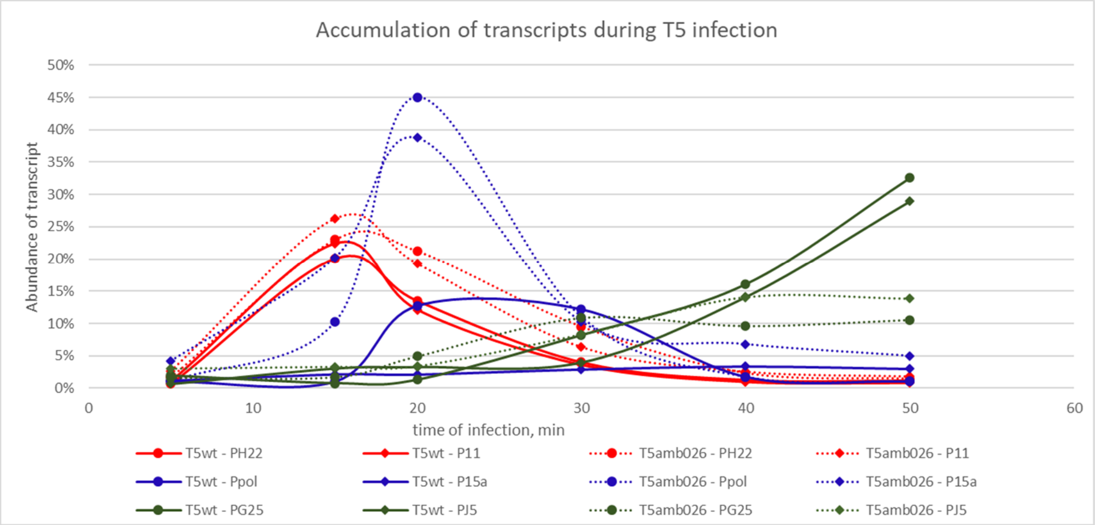

Figure S3. Quantification of indicated T5 transcripts seen on the gel shown in Figure 6 using ImageJ software. For each promoter, combined intensities of bands over all time points for wild-type or mutant phage were considered as 100%. Relative abundances (in %) at every time point for each transcript are presented.

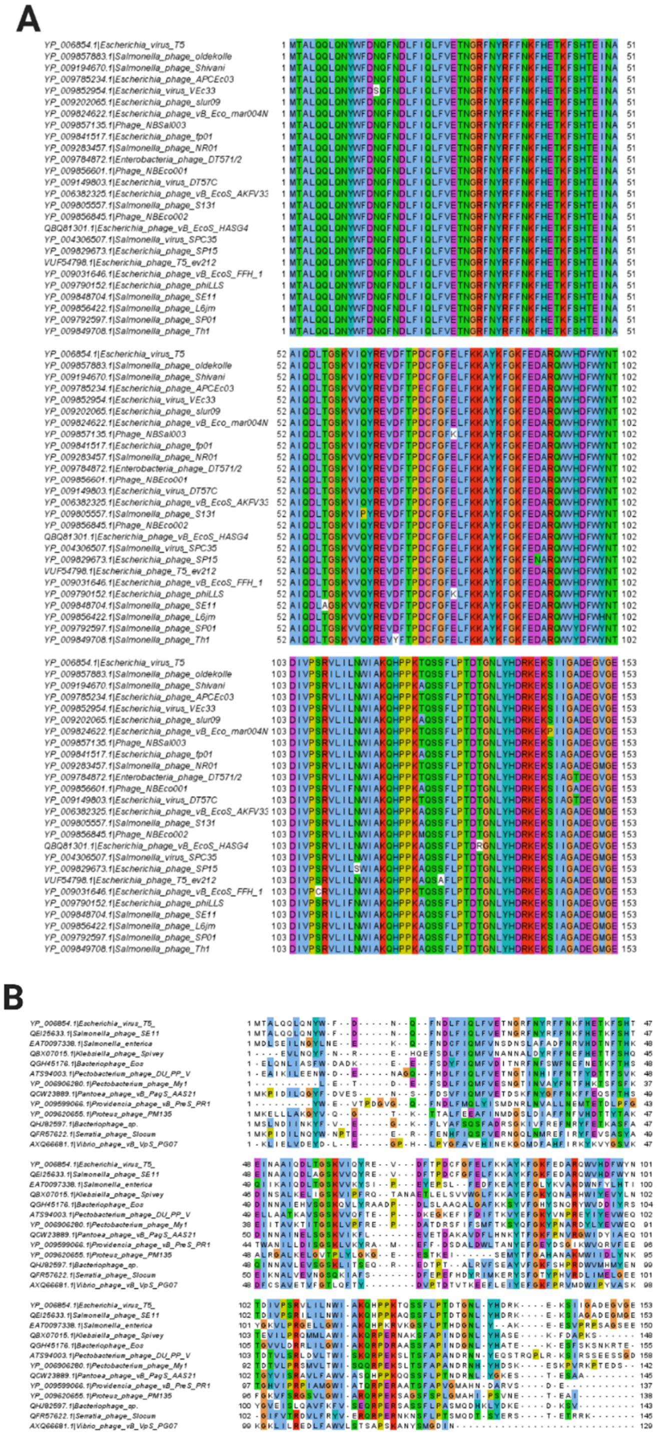

Figure S4. Conservation of gpT5.026 among phages of the *Tequintavirus* (T5-like) genus (**A**) and the *Demerecviridae* family (**B**).

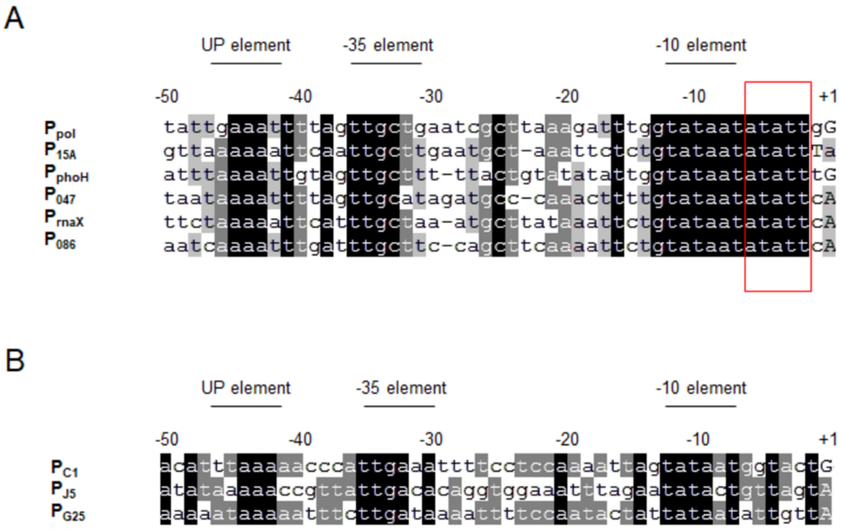

Figure S5. Multiple sequence alignment of early (**A**) and late (**B**) promoters of phage T5. Nucleotides are numbered relative to the transcription start point (+1). Likely −10 and −35 promoter elements [45], as well as an UP element, are shown. The ATATT element of early promoters is marked with a red rectangle.
